# Infected percutaneous transhepatic cholangiography catheter and rescue hepaticogastrostomy in patient with gallbladder carcinoma

**DOI:** 10.1055/a-2241-1656

**Published:** 2024-02-07

**Authors:** Sevil Tokdemir Sisman, Can Boynukara, Gurhan Sisman

**Affiliations:** 1 Department of Radiology, Seyrantepe Hamidiye Etfal Training and Research Hospital, Istanbul, Turkey; 2 Department of Internal Medicine, Acibadem Altunizade Hospital, Istanbul, Turkey; 3 162328 Department of Internal Medicine, Division of Gastroenterology, Acibadem Mehmet Ali Aydinlar University, Istanbul, Turkey

We present the case of a 58-year-old man with stage IV gallbladder carcinoma. The metastases of the liver caused obstructive jaundice, and due to the choledochal, cystic, and intrahepatic biliary duct metastases, endoscopic retrograde cholangiopancreatography (ERCP) was unsuccessful. Therefore, percutaneous transhepatic cholangiography (PTC) was performed.

The patient attended the oncology clinic because he had experienced several suppurative cholangitis episodes in the previous 4 months. The decision to remove the PTC catheter was related to erythema, swelling, and green-bluish suppurative biliary discharge due to *Pseudomonas* infection.

The computed tomography (CT) scan following intravenous contrast administration revealed multiple biliary tree metastases and an enlarged left intrahepatic biliary duct. Endoscopic ultrasound (EUS) (GF-UCT160-OL5, Olympus, Tokyo, Japan) guided the hepaticogastrostomy procedure, which was started in the biliary duct in segment II. After confirming the insertion of the biliary tree with bile drainage in the injector, a path was created, aided by a 6 Fr cystotome (Endo-Flex GmbH, Voerda, Germany), and a 7 Fr × 10 cm biliary pigtail stent (Endo-Flex GmbH, Voerda, Germany) was placed. The procedure was successful ( Video 1 ). The PTC catheter was removed after the procedure, and adequate antibiotic therapy with meropenem and vancomycin was prescribed for a week.

Endoscopic ultrasound-guided hepaticogastrostomy for rescuing an infected percutaneous transhepatic cholangiography catheter.Video 1

The biliocutaneous fistula from the PTC catheter line spontaneously closed 1 week after removal of the catheter. No complications related to hepaticogastrostomy or jaundice were observed during the 2-month follow-up period.

If the standard ERCP fails, especially for malignant diseases, EUS-guided puncture from the stomach to the biliary ducts can be performed for biliary drainage 1 2 . PTC could also be performed, although it increases susceptibility to infection. The biliocutaneous tracts associated with the infected PTC line spontaneously closed after antibiotic treatment and adequate biliary drainage from the hepaticogastrostomy 3 .

Endoscopy_UCTN_Code_TTT_1AS_2AD

## Notes

Endoscopy E-Videos https://eref.thieme.de/e-videos
*E-Videos* is an open access online section of the journal *Endoscopy* , reporting on interesting cases and new techniques in gastroenterological endoscopy. All papers include a high-quality video and are published with a Creative Commons CC-BY license. Endoscopy E-Videos qualify for HINARI discounts and waivers and eligibility is automatically checked during the submission process. We grant 100% waivers to articles whose corresponding authors are based in Group A countries and 50% waivers to those who are based in Group B countries as classified by Research4Life (see: https://www.research4life.org/access/eligibility/ ). This section has its own submission website at https://mc.manuscriptcentral.com/e-videos .
